# Safety of Tenofovir Disoproxil Fumarate–Based Antiretroviral Therapy Regimens in Pregnancy for HIV-Infected Women and Their Infants: A Systematic Review and Meta-Analysis

**DOI:** 10.1097/QAI.0000000000001359

**Published:** 2017-09-01

**Authors:** Jean B. Nachega, Olalekan A. Uthman, Lynne M. Mofenson, Jean R. Anderson, Steve Kanters, Francoise Renaud, Nathan Ford, Shaffiq Essajee, Meg C. Doherty, Edward J. Mills

**Affiliations:** * Departments of Epidemiology, Microbiology and Infectious Diseases, University of Pittsburgh, Pittsburgh, PA;; † Department of Medicine, Centre for Infectious Diseases, Stellenbosch University, Cape Town, South Africa;; ‡ Departments of Epidemiology and International Health, Johns Hopkins University, Baltimore, MD;; § Centre for Applied Health Research & Delivery, Warwick Medical School, The University of Warwick, Coventry, United Kingdom;; ‖ Department of Public Health (IHCAR), Karolinska Institute, Stockholm, Sweden;; ¶ Department of Global Health, Centre for Evidence-Based Health Care, Stellenbosch University, Tygerberg, South Africa;; # Elizabeth Glazer Pediatric AIDS Foundation, Washington, DC;; ** Department of Obstetrics and Gynecology, Johns Hopkins School of Medicine, Baltimore, MD;; †† Department of Statistics, University of British Columbia, Vancouver, Canada;; ‡‡ Global Evaluative Science, Vancouver, Canada; and; §§ Department of HIV, World Health Organization, Geneva, Switzerland.

**Keywords:** pregnancy, HIV, tenofovir, toxicity

## Abstract

**Background:**

There are limited data on adverse effects of tenofovir disoproxil fumarate (TDF)-based antiretroviral therapy (ART) on pregnant women and their infants.

**Methods:**

We conducted a systematic review of studies published between January 1980 and January 2017 that compared adverse outcomes in HIV-infected women receiving TDF- vs. non–TDF-based ART during pregnancy. The risk ratio (RR) for associations was pooled using a fixed-effects model.

**Results:**

Seventeen studies met the study inclusion criteria. We found that the rate of preterm (<37 weeks gestation) delivery (RR = 0.90, 95% confidence interval [CI]: 0.81 to 0.99, I^2^ = 59%) and stillbirth (RR = 0.60, 95% CI: 0.43 to 0.84, I^2^ = 72.0%) were significantly lower in women exposed (vs. not) to TDF-based ART regimen. We found no increased risk in maternal severe (grade 3) or potentially life-threatening (grade 4) adverse events (RR = 0.62; 95% CI: 0.30 to 1.29), miscarriage (RR = 1.09; 95% CI: 0.80 to 1.48), very preterm (<34 weeks gestation) delivery (RR = 1.08, 95% CI: 0.72 to 1.62), small for gestational age (RR = 0.87, 95% CI: 0.67 to 1.13), low birth weight (RR = 0.91; 95% CI: 0.80 to 1.04), very low birth weight (RR = 3.18; 95% CI: 0.65 to 15.63), congenital anomalies (RR = 1.03; 95% CI: 0.83 to 1.28), infant adverse outcomes or infant mortality (age >14 days) (RR = 0.65; 95% CI: 0.23 to 1.85), but increased neonatal mortality (age <14 days) risk (RR = 5.64, 95% CI: 1.70 to 18.79) with TDR-based ART exposure. No differences were found for anthropomorphic parameters at birth; one study reported minor differences in z-scores for length and head circumference at age 1 year.

**Conclusions:**

TDF-based ART in pregnancy seems generally safe for women and their infants. However, data remain limited and further studies are needed, particularly to assess neonatal mortality and infant growth/bone effects.

## Abstract 2

Supplemental Digital Content is Available in the Text.

## INTRODUCTION

The current World Health Organization (WHO) guidelines recommend antiretroviral therapy (ART) administration to all pregnant HIV-infected women, regardless of clinical or immune status, for maternal health benefits and prevention of mother-to-child HIV transmission, with tenofovir disoproxil fumarate (TDF) + lamivudine (3 TC) or emtricitabine (FTC) + efavirenz (EFV) as a fixed-dose combination, once-daily tablet,^1^ as the recommended first-line ART regimen. TDF has a good safety profile for treatment of HIV infection and is also active against hepatitis B. However, chronic TDF treatment has been associated with renal tubular dysfunction and decreases in bone mineral density in adults and young children with HIV infection as well as in adults taking TDF as pre-exposure prophylaxis.^2–8^

Data on the use of TDF-based ART in human pregnancy for treatment of HIV or hepatitis B have been generally reassuring regarding maternal toxicity and lack of association with birth defects.^9,10^ Pharmacokinetic studies indicate a slight increase in clearance of tenofovir, the active form of TDF that circulates in the bloodstream, in pregnant compared with nonpregnant women, but decreased exposure is not associated with viral rebound and standard dosing during pregnancy is recommended.^11–13^ Tenofovir readily crosses the placenta, with cord-to-maternal blood ratio ranging from 0.60 to 1.03 with chronic dosing and 0.55–0.73 with single-dose TDF in labor.^12,14,15^ Subcutaneous administration of TDF at high doses to pregnant monkeys (exposure equivalent to 25-times the area under the curve achieved with therapeutic dosing in humans) resulted in lower fetal circulating insulin-like growth factor–1, higher insulin-like growth factor binding protein–3 levels, lower fetal body weight, and a slight reduction in fetal bone porosity.^16^ Data on the effects of fetal tenofovir exposure on growth and bone development in the infant have been limited and conflicting, with some studies suggesting an effect of in utero tenofovir exposure on fetal/infant growth/bone, and others showing no effect.^17–20^

The WHO recommendation to initiate all HIV-infected pregnant women on ART irrespective of CD4^+^ cell count has been adopted by almost all countries, leading to a rapid increase in the number of pregnant women receiving a TDF-containing regimen. This systematic review meta-analysis was conducted to inform the process of revising the WHO consolidated guidelines on the use of ART for treating and preventing HIV infection. We aimed to compare rates of maternal and child adverse outcomes in pregnant HIV-infected women receiving TDF-based ART compared with those not receiving TDF-based ART.

## METHODS

### Protocol and Registration

The study background, rationale, and methods were specified in advance and documented in a protocol to be published at the international prospective register of systematic reviews (PROSPERO; Number: CRD42015025189; http://www.crd.york.ac.uk/PROSPERO/).

### Study Eligibility Criteria

Inclusion criteria were prospective cohort studies or trials that compared rates of maternal and child adverse outcomes in HIV-infected women receiving TDF-based ART compared with those not receiving TDF-based ART during pregnancy, published in any language and from any geographical regions.

### Data Sources and Searches

We conducted comprehensive systematic searches of the literature using the following databases (from inception to January 2017): Cochrane Central Register of Controlled Trials, EMBASE, LILACS, MEDLINE, TOXLINE, Web of Knowledge, WHO Global Index Medicus, and trials in progress (International Clinical Trials Registry Platform). We used keywords related to HIV, pregnancy, and TDF. We also searched conference abstracts of major HIV/AIDS conferences (conference abstracts were restricted to the last 3 years).

### Study Selection and Data Extraction

Two investigators (OU and JBN), working independently, scanned all abstracts and proceedings identified in the literature search and reviewed potentially relevant abstracts and proceedings in full text. They also extracted data on study characteristics, interventions, patient characteristics at baseline, and outcomes for the study populations of interest for the final list of selected eligible studies. Any discrepancies between the authors were resolved through discussion until a consensus was reached. We assessed the quality of evidence for the primary outcomes using the GRADE approach.^21^

### Data Synthesis

We conducted a fixed-effect meta-analysis (for outcomes with 2 or more studies) to generate pooled estimates for the associations of risk of maternal and child adverse outcomes with exposure to TDF-based ART regimens during pregnancy. Fixed-effects models are preferable when the statistical power is limited and when looking for harms associated with interventions in which the risk may be sporadically identified.^22^ Studies used the US National Institutes of Health (NIH)'s Division of AIDS (DAIDS) and the US Food and Drug Administration (FDA) Toxicity Grading System: (1) grade 1 (mild) adverse outcomes defined as transient (goes away after a short time) or mild discomfort; no limitation in activity; no medical intervention/therapy required; (2) grade 2 (moderate) adverse outcomes defined as daily activity is affected mild to moderate—some assistance might be needed; no or minimal medical intervention/therapy required; (3) grade 3 (severe) adverse outcomes defined as daily activity is markedly reduced—some assistance usually required; medical intervention/therapy required, hospitalization or hospice care possible; and grade 4 (potentially life threatening) adverse outcomes with extreme limitation to daily activity, significant assistance required; significant medical intervention/therapy, hospitalization or hospice care very likely.^23^ Results were presented as pooled risk ratio (RRs) with 95% confidence intervals (CIs). Data from trials and observational cohorts were pooled together given the lack of difference in average risk estimates between study types.^24^ Heterogeneity was quantified using the I^2^ statistics (the proportion of variation due to between study heterogeneity).^25^ Review Manager 5.1 was used for statistical analyses.

## RESULTS

### Study Characteristics

**FIGURE 1.**
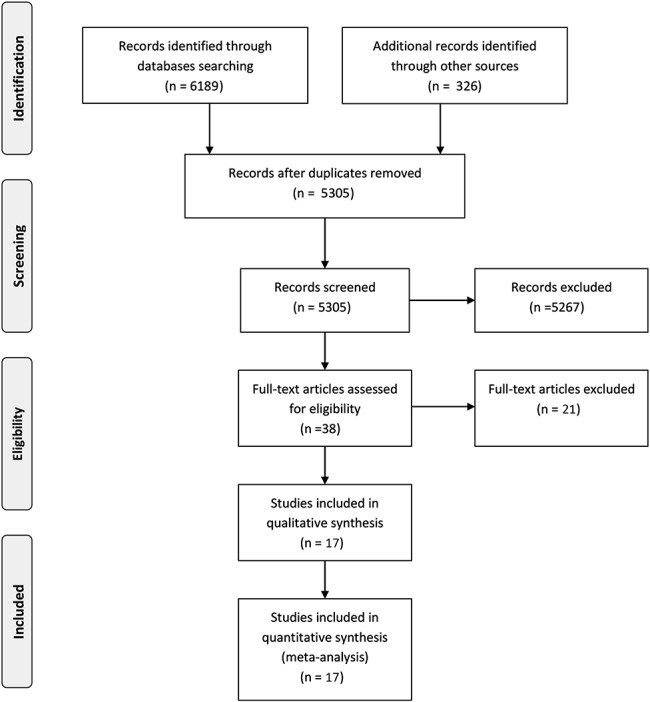
Summary of evidence search and selection.

The process of study identification and selection is shown in Figure 1. The literature search yielded 5305 citations after removing duplicates. After reviews of the title and abstract, 38 full-text articles were selected for critical reading. Seventeen studies met the inclusion criteria.^17–20,26–38^ Table 1 shows the characteristics of these studies. Most of the included studies were prospective cohorts (n = 14)^17–20,26,27,29–33,35,36,38^; 2 were from the same randomized controlled trial^34,37^ and 1 was a cross-sectional study.^28^ The studies recruited participants from 1993 to 2014 and were published between 2007 and 2016. Eight publications including 6 cohorts (with 1 cohort having 3 publications) were from the United States,^19,26,27,29–31,35,36^ and 1 each was from Botswana,^38^ Malawi,^18^ Italy,^20^ and France.^32^ Four studies were multicountry studies: 1 study recruited participants from Uganda and Zimbabwe^17^; 1 from Malawi, South Africa, Uganda, and Zimbabwe^37^; 1 from India, Malawi, South Africa, Tanzania, Uganda, Zambia, and Zimbabwe^34^; and 1 from 17 centers across European countries.^33^

**TABLE 1.**
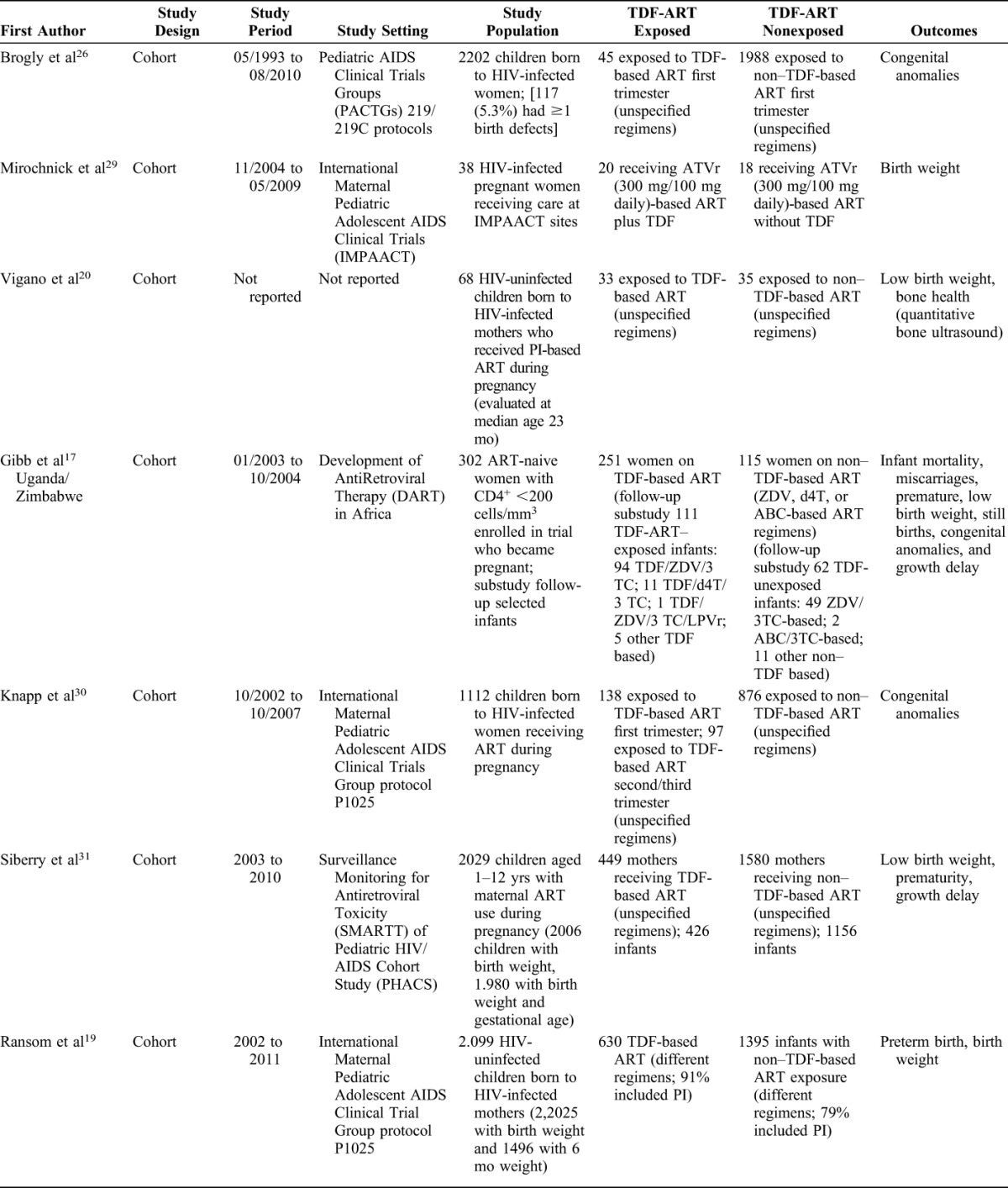

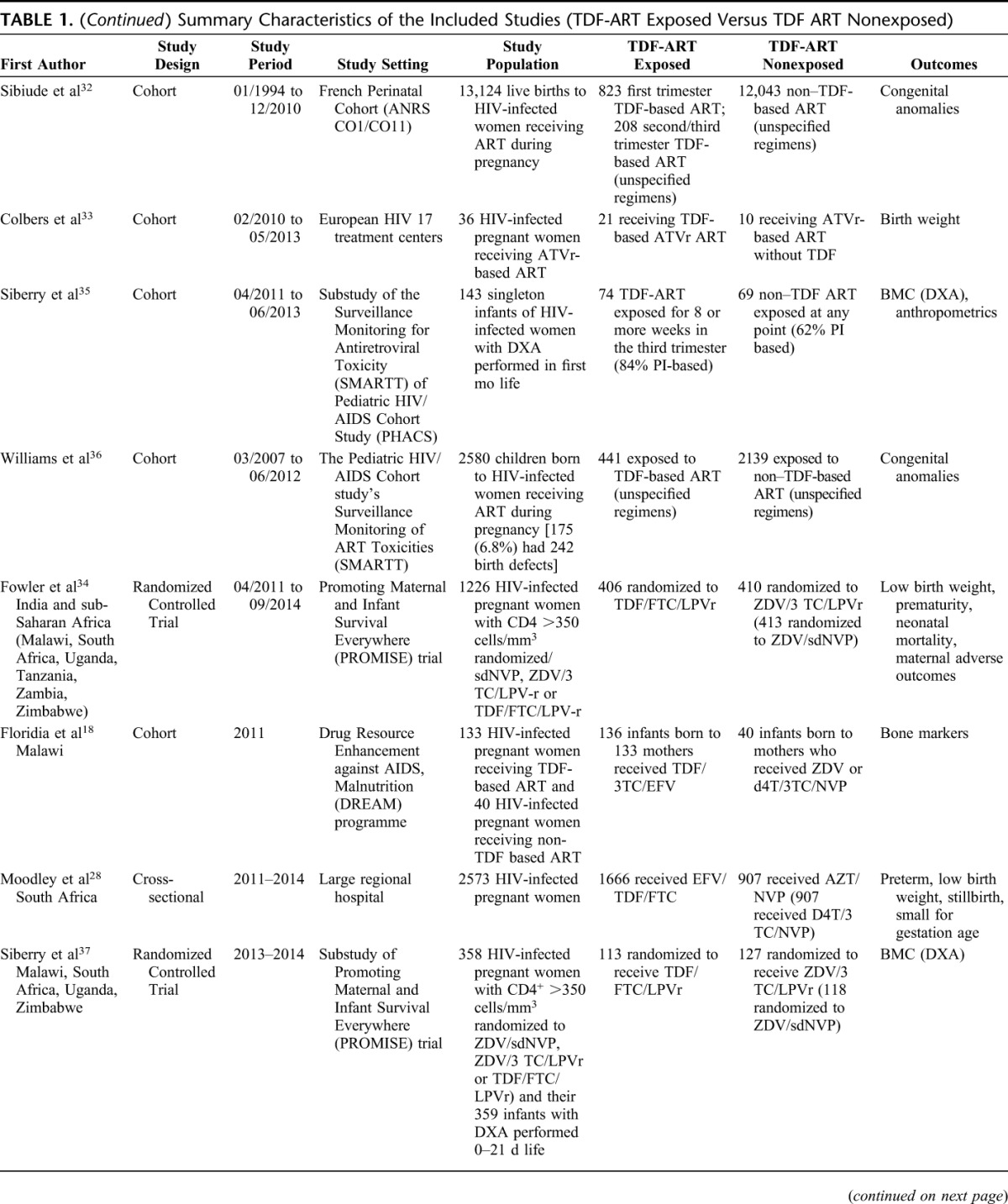

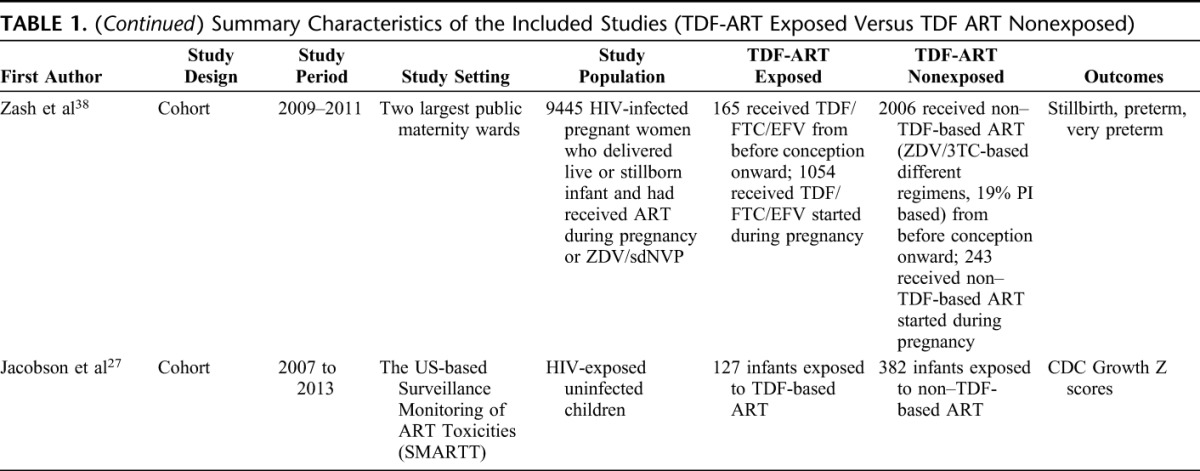
Summary Characteristics of the Included Studies (TDF-ART Exposed Versus TDF ART Nonexposed)

### Risk of Maternal and Obstetrical Adverse Outcomes

**FIGURE 2.**
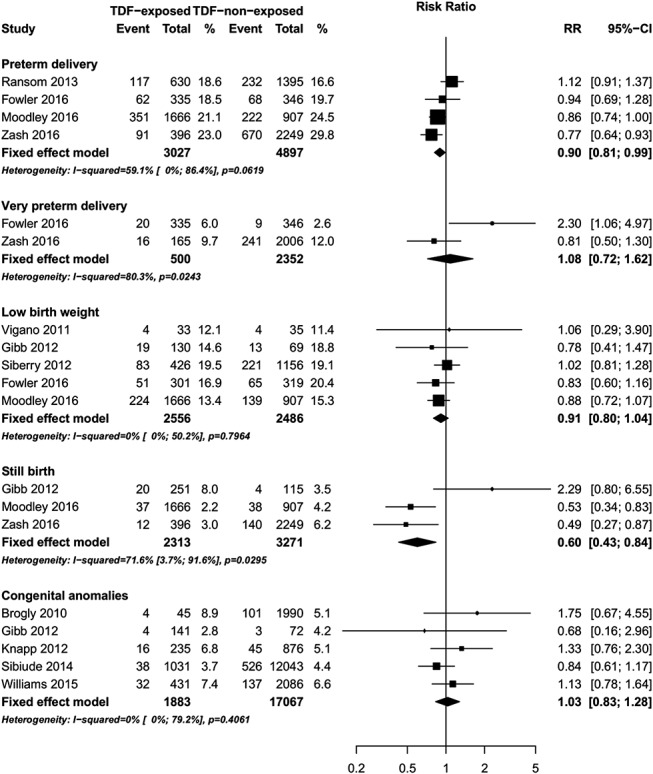
Forest plot of risk of maternal adverse outcomes (TDF exposed vs. TDF–nonexposed).

The pooled RRs for the association between receipt of TDF-based ART during pregnancy and maternal and obstetric adverse outcomes are provided in Figure 2. We found that the rate of preterm (<37-week gestation) delivery (RR = 0.90, 95% CI: 0.81 to 0.99, I^2^ = 59%, 4 studies) and stillbirth (RR = 0.60, 95% CI: 0.43 to 0.84, I^2^ = 72.0%, 3 studies) were significantly lower in women exposed to TDF-based ART compared with those exposed to non–TDF-based ART (Fig. 2). We found no increased risk in maternal grade 2 or 3 or 4) adverse events (AEs) (RR = 0.62, 95% CI: 0.30 to 1.29, 1 study), small for gestational age (RR = 0.87, 95% CI: 0.67 to 1.13, 1 study), miscarriage (RR = 1.09, 95% CI: 0.80 to 1.48, 1 study), or low birth weight (<2500 g) (pooled RR 0.91, 95% CI: 0.80 to 1.04, I^2^ = 0%, 5 studies), in women receiving TDF-based ART compared with those receiving non–TDF-based ART. Two studies reported on very preterm (<34-week gestation) delivery^34,38^ (RR = 1.08, 95% CI: 0.72 to 1.02, 2 studies); the Promoting Maternal and Infant Survival Everywhere (PROMISE) trial which was conducted at 14 sites in 7 countries (India, Malawi, South Africa, Tanzania, Uganda, Zambia, and Zimbabwe), Fowler et al, found that exposure to TDF-based ART increased the risk of very preterm delivery (RR = 2.30, 95% CI: 1.06 to 4.97),^33^ whereas in another study conducted in Botswana by Zash et al found no significant difference in the rate of very preterm delivery (RR = 0.81, 95% CI: 0.50 to 1.30) between women who received TDF-based ART and those who received non–TDF-based ART.^37^ Also, the PROMISE trial reported on very low birth weight (<1500 g); although this was higher with TDF-based ART (2%) compared with non–TDF-based ART (0.6%), this was not statistically significant (*P* = 0.17).^33^

Five studies reported congenital anomalies as an outcome^17,26,30,32,36^ (Fig. 2). The reported congenital anomalies varied between the 5 studies with no pattern in types of anomalies observed. There was no statistically significant difference in the risk of congenital anomalies between infants born to HIV-infected pregnant women receiving first-trimester TDF-based vs non–TDF-based ART (pooled RR = 1.03, 95% CI: 0.83 to 1.28, I^2^ = 0%, 5 studies); among 1883 HIV-infected pregnant women receiving first-trimester TDF-based ART, 94 gave birth to infants with congenital anomalies (5.0%, 95% CI: 4.1% to 6.1%) compared with 812 out of 17,067 (4.8%, 95% CI: 4.4% to 5.1%) who received non–TDF ART.

### Risk of Infant Adverse Outcomes

**FIGURE 3.**
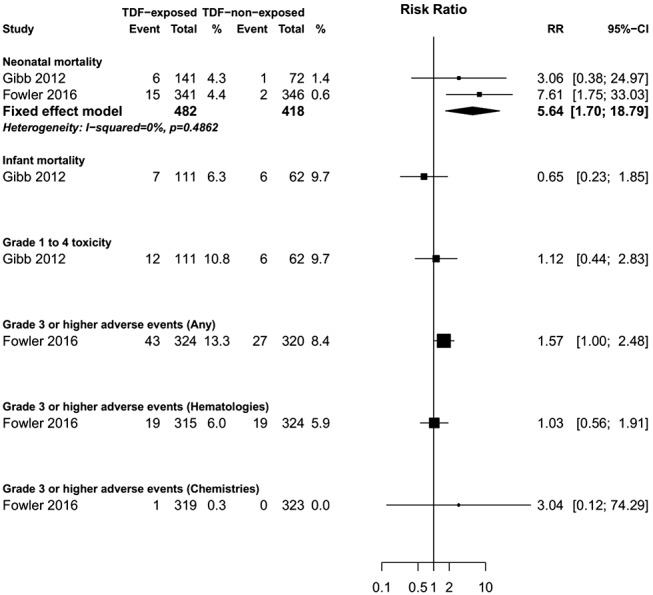
Forest plot of risk of child adverse outcomes (TDF exposed vs. TDF–nonexposed).

The pooled RRs for the association of various outcomes for infants born to mothers who received TDF-based ART vs. non–TDF-based ART during pregnancy are provided in Figure 3. No significant differences in AEs were observed in infants born to mothers who received TDF-based ART vs. non–TDF-based ART in 2 studies.^17,34^ In one study, there was no significant difference in grades 1–4 levels of creatinine, phosphorus, alkaline phosphatase, hemoglobin, platelets, or neutrophils between infants exposed to TDF ART vs. non–TDF ART.^17^ In a randomized clinical trial, no significant differences in grade 3 or 4 signs/symptoms/diagnoses or hematologic or chemistry laboratory parameters were observed between TDF-based ART vs. non–TDF-based ART-exposed infants.^34^

#### Neonatal/Infant Mortality

Two studies reported on neonatal (<14 days) mortality^17,34^ (RR = 5.64, 95% CI: 1.70 to 18.79, 2 studies, Fig. 3). The PROMISE trial by Fowler et al,^34^ randomized HIV-infected pregnant women with CD4^+^ cell count >350 cells/mm^3^ starting at 14-week gestation to either tenofovir/emtricitabine/lopinavir–ritonavir (TDF-ART), zidovudine/lamivudine/lopinavir–ritonavir (non–TDF ART), or zidovudine plus intrapartum single-dose nevirapine for prevention of mother-to-child transmission. Although this study found an increased risk in neonatal (age <14 days) mortality in infants exposed to TDF-based ART compared with those exposed to non–TDF-based ART (RR = 7.61, 95% CI: 1.75 to 33.03), there was no significant difference in neonatal mortality in infants exposed to TDF-based compared with those exposed to zidovudine/single-dose nevirapine (RR = 1.40, 95% CI: 0.65 to 2.99).^34^ The Development of AntiRetroviral Therapy in Africa (DART) trial by Gibb et al,^17^ followed Ugandan and Zimbabwean women randomized to TDF-based ART or non–TDF-based ART who became pregnant and evaluated pregnancy outcomes. This study found no difference in neonatal mortality (age <14 days) (RR 3.06, 95% CI: 0.38 to 24.97) or infant mortality (age >14 days) (RR = 0.65, 95% CI: 0.23 to 1.85, 1 study; Fig. 3).

#### Growth

**FIGURE 4.**
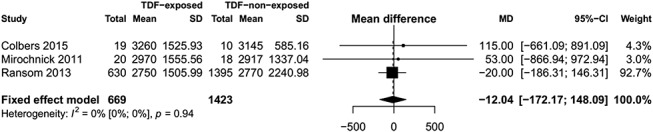
Forest plot of mean birth weight (TDF-exposed vs. TDF–nonexposed).

**FIGURE 5.**
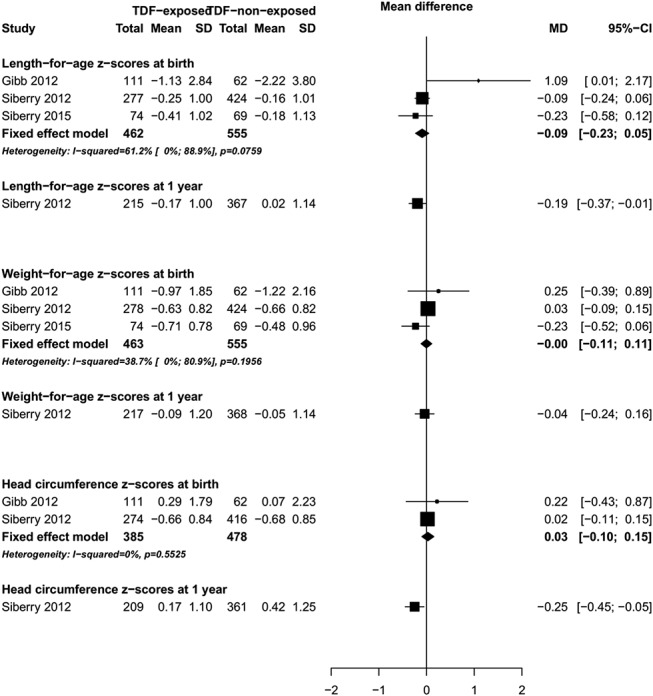
Forest plot of growth Z scores at birth (TDF-exposed vs. TDF–nonexposed).

We found no difference in mean birth weight (mean difference = −12.04 g, 95% CI: −172.17 to 148.09, I^2^ = 0%, 3 studies, Fig. 4), mean weight-for-age Z-scores at birth (mean difference = −0.00, 95% CI: −0.11 to 0.11, I^2^ = 39%, 3 studies), mean weight-for-age Z-scores at 1 year (mean difference = −0.04, 95% CI: −0.24 to 0.16, 1 study), mean length-for-age Z-scores at birth (mean difference = −0.09, 95% CI: −0.23 to 0.05, I^2^ = 61%, 3 studies), or head circumference Z-scores at birth (mean difference = 0.03, 95% CI: −0.10 to 0.15, I^2^ = 0%, 2 studies) in infants born to mothers receiving TDF-based ART compared with those born to mothers receiving non–TDF-based ART during pregnancy (Fig. 5). However, one study reported that at 1 year of age, the mean length-for-age Z-scores (mean difference = −0.19, 95% CI: −0.37 to −0.01, one study) and head circumference z-scores (mean difference = −0.25, 95% CI: −0.45 to −0.05, one study) were significantly smaller in infants born to mothers receiving TDF-based ART compared with those born to mothers receiving non–TDF-based ART during pregnancy.^31^

In one study from the United States, Jacobson et al^27^ reported growth measures at 2 years of age (Supplemental Digital Content, Figure 1 http://links.lww.com/QAI/A988). Among children born to women initiating ART in the first trimester, TDF-based ART-exposed children had slightly higher mean weight Z-scores (0.30 SD 95% CI: −0.18 to 0.78), length Z-scores (0.22 SD 95% CI: −0.19 to 0.63), weight-for-length Z-scores (0.21 SD 95% CI: −0.25 to 0.67), head circumference Z-scores (0.27 SD 95% CI: −1.08 to 1.62), and lower triceps skin fold Z-scores (−0.13 SD 95% CI: −0.57 to 0.31) compared with children exposed to non–TDF-based ART; however, none of these differences were statistically significant (Supplemental Digital Content, Figure 1 http://links.lww.com/QAI/A988). Similarly, among children born to women initiating ART in the second trimester, there were no differences in mean weight Z-scores (−0.09 SD 95% CI: 0.39 to 0.21), length Z-scores (−0.14, 95% CI: −0.41 to 0.13), weight-for-length Z-scores (−0.01 SD 95% CI: −0.33 to 0.31), head circumference Z-scores (0.01 SD 95% CI: −0.29 to 0.31), and triceps skin fold Z-scores (−0.05 SD 95% CI: −0.41 to 0.31) in children born to mothers who received TDF-based ART vs. those who received non–TDF-based ART during pregnancy (Supplemental Digital Content, Figure 1 http://links.lww.com/QAI/A988). A second study from Africa similarly noted no significant difference in weight- and height-for-age Z scores from birth through age 5 years in 111 infants born to mothers receiving TDF-based ART compared with 62 born to mothers receiving non–TDF-based ART during pregnancy.^17^

#### Bone Health

In the studies we examined, the potential effects of in utero exposure to tenofovir on infant bone health were evaluated by different methods and this precluded our ability to combine the results in a meta-analysis.

One study evaluated bone health by tibial quantitative ultrasound, finding no significant difference between infants at 23 months (median age) born to mothers receiving TDF-based ART compared with non–TDF-based ART (mean difference in tibial speed of sound −0.20, 95% CI: −2.28 to 1.88, 1study).^20^ In one study, tenofovir-unexposed and -exposed children had similar mean levels of bone markers (C-terminal telopeptide of type I collagen, CTX; and bone-specific alkaline phosphatase, BAP) at 6 months (CTX: 0.62 versus 0.55 ng/mL, *P* = 0.122; BAP: 384 versus 362 U/L, *P* = 0.631).^18^

In a substudy of a prospectively followed observational US cohort (Pediatric HIV/AIDS Cohort Study, PHACS), bone mineral content (BMC) was measured by dual-energy x-ray absorptiometry (DXA) scan within 5 weeks of birth in HIV-exposed but uninfected singleton infants born at ≥36-week gestation. DXA scan results were compared between 74 infants born to mothers who received TDF-based ART for >8 weeks in the third trimester of pregnancy to results from 69 infants born to mothers who received non–TDF-based ART during pregnancy.^35^ Significantly lower mean BMC was reported in infants whose mothers received TDF-based ART compared with non–TDF-based ART during pregnancy (12% difference, mean BMC 56 grams in infants whose mothers received TDF-based ART vs. 63.8 g in infants whose mothers received non–TDF-based ART, *P* = 0.002).

However, more recently, neonatal BMC measured by DXA scan at 0–21 days of age was evaluated in HIV-exposed infants in a substudy of a randomized clinical trial comparing different approaches to prevention of in utero perinatal HIV transmission in HIV-infected African women with CD4^+^ T-cell counts >350 cells/mm^3^ (the PROMISE trial, described earlier).^37^ Whole-body BMC was significantly lower in infants whose mothers received either TDF-based ART compared with zidovudine/single-dose nevirapine (estimated mean difference 9.73 grams, 95% CI: 5.49 to 13.96, *P* < 0.001) or non–TDF-based ART compared with zidovudine/single-dose nevirapine (estimated mean difference 7.97 g, 95% CI: 3.97 to 11.96, *P* < 0.001). However, there was no significant difference between infants exposed to TDF-based ART vs. those on non–TDF-based ART (estimated mean difference 1.76 g, 95% CI: −2.43 to 5.95, *P* = 0.41).^37^ No significant difference was seen between any of the study arms when lumbar spine BMC measurements were compared.

#### Quality of the Evidence

Our assessment of the quality of evidence using the GRADE approach is shown in Table 2. The quality of the evidence was judged low (low birth weight and congenital abnormalities) to very low (prematurity, birth weight, miscarriage, stillbirth, neonatal mortality, and infant mortality), and therefore further research is needed.

**TABLE 2.**
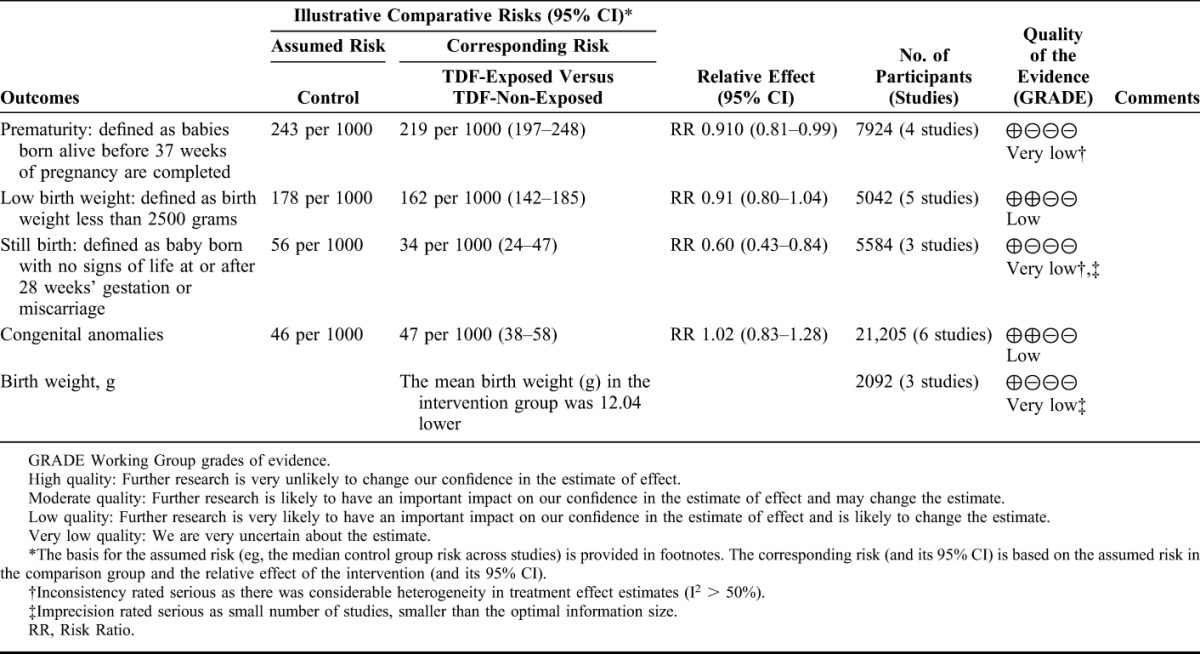
GRADE Summary of Finding

## DISCUSSION

Reassuringly, we found no statistically significant differences between HIV-infected pregnant women receiving TDF-based vs those receiving non–TDF-based ART in the risks of maternal or infant grade 3 or 4 adverse outcomes, pregnancy loss or miscarriage, small for gestational age, low birth weight, congenital anomalies, or infant mortality at age >14 days. Rates of preterm delivery (<37-week gestation) and stillbirth were modestly lower in women receiving TDF-based compared with those receiving non–TDF-based ART. Also a recent study from Malawi on pregnancy outcomes with Option B+, in which women receive TDF-efavirenz-based ART, found that risk of very preterm birth was 2.3 times higher among women not receiving ART than those receiving TDF-based ART^39^; furthermore, in a study from Botswana, TDF-based ART initiated during pregnancy was associated with lower rates small for gestational age infants than those on non–TDF ART, but similar rates of preterm delivery and stillbirth.^38^ However, outside congenital anomalies, available data on the effects of TDF-containing ART in pregnancy remain relatively limited.

Our data on congenital anomalies are consistent with data from the Antiretroviral Pregnancy Registry. The registry has recorded sufficient numbers of first-trimester TDF exposures to confidently rule out 1.5-fold increased risk of overall birth defects, with a prevalence of birth defects with first-trimester TDF exposure of 2.3% as compared to 2.7% total prevalence in the US population.^9^ To date, available human data suggest that the use of TDF-based ART during pregnancy does not increase the risk of major congenital anomalies.

One study, the PROMISE trial, reported elevated rates of neonatal (age <14 days) mortality in infants born to mothers receiving TDF-based ART compared with those born to mothers receiving non–TDF-based ART.^34^ This study enrolled HIV-infected pregnant women with CD4^+^ counts >350 cells/mm^3^ and randomized them to receive zidovudine alone plus intrapartum single-dose nevirapine, TDF-based ART (TDF/emtricitabine/lopinavir–ritonavir), or non–TDF-based ART (zidovudine/lamivudine/lopinavir–ritonavir). In the initial 1½ years of enrollment (during which 65% of participants enrolled), only women with hepatitis B virus coinfection were allowed to be randomized to the TDF ART arm, but after a protocol modification, in the second 1½ years of enrollment (accounting for 35% of enrollment), all HIV-infected women regardless of hepatitis B status were randomized among all 3 arms. TDF ART comparisons were limited to the second part of the study with concomitant randomization to all arms. There were no significant differences between the TDF ART and non–TDF ART arms in maternal grade ≥2 AEs, spontaneous abortion, stillbirth, preterm delivery <37 weeks, low birth weight <2500 grams, very low birth weight <1500 grams, or grade ≥3 infant AEs between infants born to mothers in the TDF ART and non–TDF ART arms. However, there was a lower rate of very preterm delivery (<34 weeks) in the non–TDF ART compared with TDF ART arm (2.6% vs. 6.0%, respectively, *P* = 0.04), which led to a difference in neonatal mortality (age <14 days) (0.6% with non–TDF ART vs. 4.4% with TDF ART, *P* = 0.001), as most deaths were among very preterm infants. It is important to note, however, that there was not a significant difference between the TDF ART arm and zidovudine/single-dose nevirapine arm in very preterm delivery (6.0% vs. 3.2%, respectively, *P* = 0.10) or neonatal mortality (4.4% vs. 3.2%, *P* = 0.43). In addition, of the 17 neonatal deaths in the non–TDF ART arm, 88% (N = 15) occurred during the initial period of the trial and only 12% (N = 2) during the period of comparison with TDF ART. This suggests that the non–TDF ART arm may have had artificially low rates of very preterm delivery and infant mortality during the 3-arm comparative period of the study, and not that the TDF-ART arm had elevated risk of these events.

In contrast to the PROMISE trial results, rates of very preterm delivery were similar to TDF ART (combined with efavirenz) compared with non–TDF ART exposure in a Botswana study by Zash et al,^38^ and no differences in neonatal mortality were observed with TDF ART exposure (primarily combined with nevirapine) compared with non–TDF ART exposure in a study in Uganda/Zimbabwe.^17^ It is important to note that the PROMISE trial used lopinavir, a protease inhibitor (PI) that is coformulated with ritonavir (LPV/r)-based ART regimens, whereas the Botswana and Uganda/Zimbabwe studies use non–PI-based ART regimens. In addition, in the PROMISE trial, the LPV/r dose was increased during the third trimester, based on studies showing decreased lopinavir–ritonavir levels with standard doses in pregnancy.^40^ A pharmacokinetic interaction has been reported with concurrent administration of tenofovir and lopinavir–ritonavir, which could result in increased plasma and intracellular tenofovir levels.^41,42^ Of note, the WHO-recommended first-line ART regimen for HIV-infected pregnant women [TDF + FTC (or 3 TC) + EFV] is distinct from the LPV/r-based ART regimen used in the PROMISE study. The results from other studies have not suggested that TDF is associated with excess adverse outcomes. By contrast, PIs, including LPV/r, have been reported to be associated with prematurity and low birth weight.^43–45^ As LPV/r-based regimen is recommended for second-line ART regimen,^1^ toxicity associated with such ART regimens needs further research. The PROMISE team is evaluating this further.

Data on the effects of in utero tenofovir exposure on bone and long-term growth are inadequate and therefore we cannot draw any conclusions at this time. One study that evaluated bone density using tibial quantitative ultrasound at a median age of 23 months found no significant difference in tibial speed of sound measurement between HIV-exposed uninfected children born to mothers who received TDF-based ART during pregnancy and those whose mothers received non–TDF ART.^19^ However, DXA scan is considered the gold standard for bone mineral status assessments.^46^ In a substudy within the observational PHACS cohort which assessed BMC by DXA scan in HIV-exposed uninfected infants aged <5 weeks, lower BMC was observed in infants born to mothers receiving TDF ART compared with those receiving non–TDF ART; however, observational studies are subject to potential unknown confounders and longitudinal data are not available to determine whether this persists or if it has any clinical significance to the growing child no longer exposed to tenofovir. In contrast, the PROMISE trial substudy reported lower BMC in neonates exposed to both TDF-based and zidovudine-based ART compared with those exposed to zidovudine/single-dose nevirapine, but there were not significant differences between BMC when comparing infants exposed to TDF-based ART vs. zidovudine-based ART. However, we should note that in this study there was higher neonatal mortality in the TDF arm; therefore, it is possible that some infants affected by TDF exposure did not survive long enough to have a DXA scan (survival bias).

Available data do not indicate any difference in anthropometric measurements at birth with in utero tenofovir exposure, but there are conflicting data on whether there might be some delayed effects at age 6 months–1 year.^17,31^ The biologic plausibility of later emergence of growth effects due to in utero tenofovir exposure, especially when they are not observed at birth and there is not continued exposure (studies analyzed were in formula-fed populations), is unclear. Again, there are limited longitudinal data to determine whether such findings persist or have clinical relevance; 2 studies with longer follow-up (to age 2–5 years) did not find any differences in growth with TDF exposure. More data in larger cohorts are needed to be able to determine whether there are any adverse effects of in utero tenofovir exposure on bone or long-term growth.

Strengths of our meta-analysis include use of a standard protocol, a comprehensive and updated literature search strategy, and involvement of 2 independent reviewers in all stages of the review process. There are also limitations of the study that should be considered. First, some of the studies had small sample sizes. Second, the review was limited by the small number of studies reporting most of the outcomes of interest. Most of the pooled estimates should be interpreted with caution, and more research with larger sample sizes is needed to confirm these findings. We applied the I^2^ value to assess heterogeneity. It is worth considering that I^2^ values are typically high, and potentially exaggerated, when combining observational studies; therefore, interpreting the I^2^ values should be performed with caution.^47^ Finally, inclusion of observational studies may lead to bias because unknown confounding that were not adjusted for, other than ART, might be responsible for maternal or infant AEs.

In summary, although the available data suggest that use of a TDF-containing ART regimen seems to be safe for HIV-infected pregnant women and their infants, the data remain limited and few studies addressed maternal toxicity or infant growth and bone effects. In consideration of these data, the good safety and efficacy data of TDF-containing first-line therapy overall, and the programmatic advantages of harmonizing ART across populations, the latest WHO guidelines continue to recommend TDF + FTC (or 3 TC) + EFV as first-line ART for adults, including pregnant women. Nevertheless, given the expected global increase in TDF use with implementation of WHO guidelines for universal treatment for all HIV-infected individuals, including pregnant and nonpregnant women, additional research on the safety of TDF in pregnancy is needed, particularly prospective longitudinal data on growth and maternal/infant bone density measurements.

## Supplementary Material

SUPPLEMENTARY MATERIAL
