# Supplementary material for: Safety of Tenofovir Disoproxil Fumarate–Based Antiretroviral Therapy Regimens in Pregnancy for HIV-Infected Women and Their Infants: A Systematic Review and Meta-Analysis
Source: J Acquir Immune Defic Syndr. 2017 Sep 1;76(1):1–12. doi: 10.1097/QAI.0000000000001359 (PMC5553236; doi:10.1097/QAI.0000000000001359)
Supplement: SUPPLEMENTARY MATERIAL [file qai-76-01-s001.docx]

**Supplementary materials**

**eFigure 1: Forest plot of CDC growth Z scores, at two years of age, Jacobson et al. 2017**[**^27^**](#_ENREF_27)

**
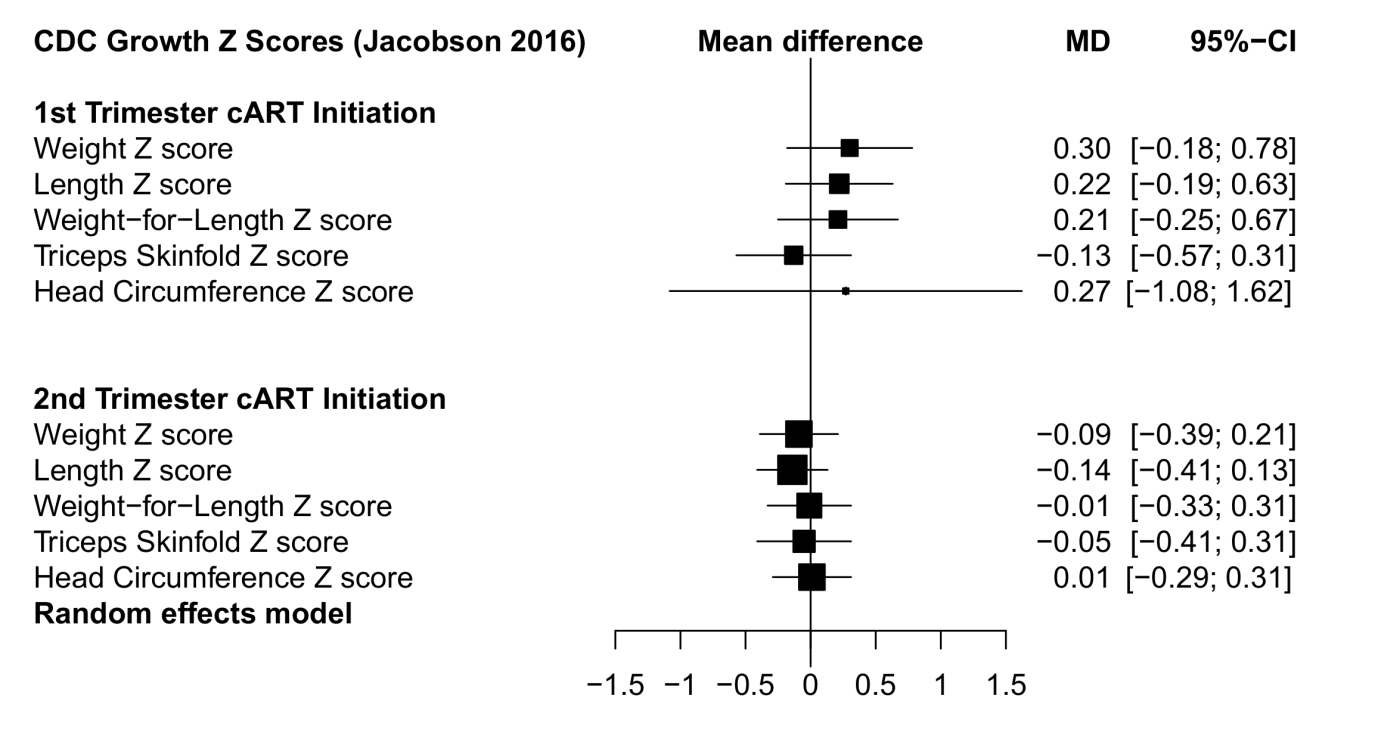
**
